# Exploiting autophagy balance in T and NK cells as a new strategy to implement adoptive cell therapies

**DOI:** 10.1186/s12943-023-01893-w

**Published:** 2023-12-09

**Authors:** Manuela Giansanti, Tobias Theinert, Sarah Katharina Boeing, Dorothee Haas, Paul-Gerhardt Schlegel, Paola Vacca, Francesca Nazio, Ignazio Caruana

**Affiliations:** 1 Immunology Research Area, Innate Lymphoid Cells Unit, Bambino Gesù Children’s Hospital (IRCCS), Rome, Italy; 2 Department of Pediatric Hematology, Oncology and Stem Cell Transplantation, University Hospital Würzburg, 97080 Würzburg, Germany; 3 Department of Biology, University of Rome Tor Vergata, 00133 Rome, Italy

**Keywords:** Autophagy, Effector cells, Mitophagy, Metabolism, T and NK development

## Abstract

Autophagy is an essential cellular homeostasis pathway initiated by multiple stimuli ranging from nutrient deprivation to viral infection, playing a key role in human health and disease. At present, a growing number of evidence suggests a role of autophagy as a primitive innate immune form of defense for eukaryotic cells, interacting with components of innate immune signaling pathways and regulating thymic selection, antigen presentation, cytokine production and T/NK cell homeostasis. In cancer, autophagy is intimately involved in the immunological control of tumor progression and response to therapy. However, very little is known about the role and impact of autophagy in T and NK cells, the main players in the active fight against infections and tumors. Important questions are emerging: what role does autophagy play on T/NK cells? Could its modulation lead to any advantages? Could specific targeting of autophagy on tumor cells (blocking) and T/NK cells (activation) be a new intervention strategy? In this review, we debate preclinical studies that have identified autophagy as a key regulator of immune responses by modulating the functions of different immune cells and discuss the redundancy or diversity among the subpopulations of both T and NK cells in physiologic context and in cancer.

## Introduction

**Fig. 1:**
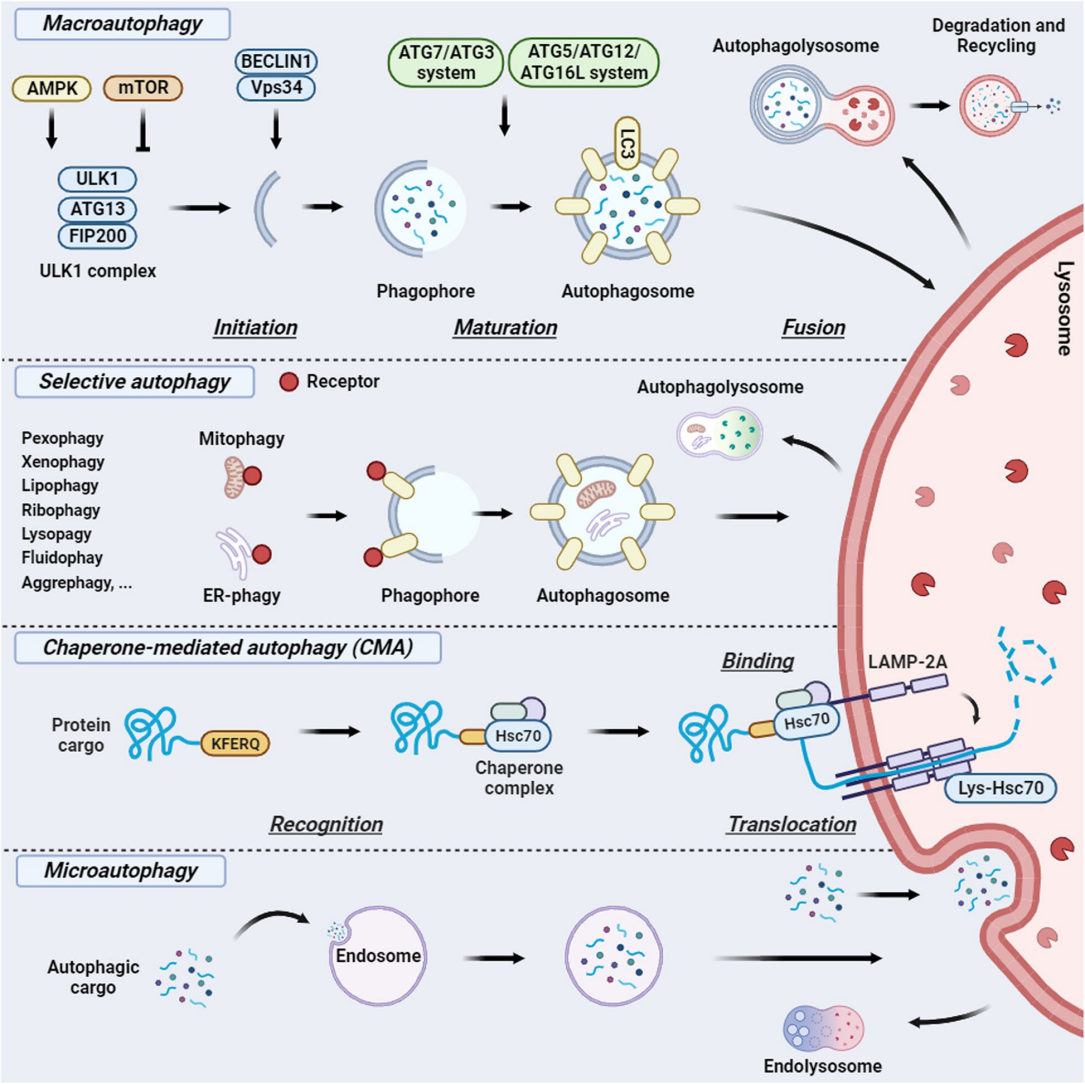
Primary types of autophagy: a schematic overview. Macroautophagy involves the regulation of Unc-51-like kinase 1 (ULK1) complex by both AMPK and mTOR. This complex mediates the initial stage of double-membrane scaffold formation around the autophagy cargo by recruiting the Beclin1-Vps34 complex in the proximity of the phagophore. In the next step of elongation, ATG7-ATG3 and ATG5-ATG12-ATG6L1 complexes mediate the conjugation of lipidated microtubule-associated protein 1A/1B light chain 3 (LC3I) family members with phosphatidylethanolamine (LC3II). Mature autophagosomes fuse with lysosome to form the autophagolysosome, in which autophagic cargo is degraded and recycled. Selective autophagy leads to the degradation of damaged organelles (e.g., pexophagy, ribophagy, lysophagy) or substrates (e.g. aggrephagy, fluidophagy, lipophagy) through the binding of selective receptors with autophagy core proteins (LC3B, GABARAP). Chaperone-mediated autophagy (CMA) is a form of autophagy in which cytoplasmic KFERQ motif proteins are recruited by the Hsc70 chaperone complex. It, then, binds to the lysosome-associated membrane protein type 2 isoform A (LAMP-2A) on the lysosome membrane, which undergoes oligomerization to form a transport channer that mediates the translocation of protein cargo into lysosome for its degradation. Microautophagy, instead, mediates a non-selective up-take of cytoplasmic cargo through invagination (or protrusion) of lysosome membrane. The figure is created with “BioRender.com”

Autophagy is a highly conserved degradation process occurring in all eukaryotic cells to maintain homeostasis and cell survival during development and in response to stressful conditions [1, 2]. Autophagy is also involved in removing specifically damaged or dysfunctional organelles such as mitochondria (by a process called mitophagy), endoplasmic reticulum (ER-phagy) as well as degrading intracellular pathogens (xenophagy) [3]. Three main types of autophagy have been reported: microautophagy, macroautophagy and chaperone-mediated autophagy (CMA). Microautophagy is a non-selective lysosomal degradative process, involving direct engulfment of cytoplasmic cargo at a boundary membrane by autophagic tubes. Macroautophagy, (hereafter referred to as autophagy) sequesters portions of the cytoplasm or organelles by double membrane vesicles called autophagosomes and then deliversed to lysosomes for degradation. CMA refers to the chaperone-dependent selection of soluble cytosolic proteins that are then targeted to lysosomes. At the core of the molecular machinery of autophagy is a specific group of genes called *ATG* [2, 4]*.* The initiation step of the autophagy process involves the activation of Unc-51-like kinase (ULK) complex, comprising ULK1 kinase, ATG13 and focal adhesion kinase interacting protein 200 kDa (FIP200). This protein complex is inhibited by the master cell growth regulator, the mammalian target of rapamycin (mTOR) and activated by the major sensor of energy stress, AMP-activated protein kinase (AMPK). After ULK1 complex activation, the Beclin1-VPS34 complex is recruited to the phagophore, a membrane platform near the endoplasmic reticulum (ER) which then expands to capture cytoplasmic materials becoming the double-membrane autophagosome. This phase of elongation is regulated by two protein conjugation systems ATG7-ATG3 and the ATG5-ATG12-ATG6L1 complex that mediate the conjugation of lipidated microtubule-associated protein 1A/1B light chain 3 (LC3I) family members to phosphatidylethanolamine (PE) (LC3II). This event is crucial for specific substrate recognition in the selective degradation process. Additionally, a substantial number of selective autophagy receptors (such as CALCOCO2 and OPTN or BCL2 interacting protein 3 (BNIP3) and BCL2 interacting protein 3 like (BNIP3L)) have also been identified that mediate the binding of the cargo material and the autophagosomal membrane (Fig. 1) [5].

At present, there is a growing amount of evidence on how autophagy and its related processes have an impact on both T and Natural killer (NK) cell differentiation, development, homeostasis, metabolic regulation, function and survival [6, 7]. In this review, we summarize the recent preclinical studies that have identified autophagy-related mechanisms in controlling T and NK cell biology and immunogenicity in cancer, discussing possible strategies for enhancing T and NK cellular mediated-therapies.

## Autophagy during T cell differentiation

**Fig. 2:**
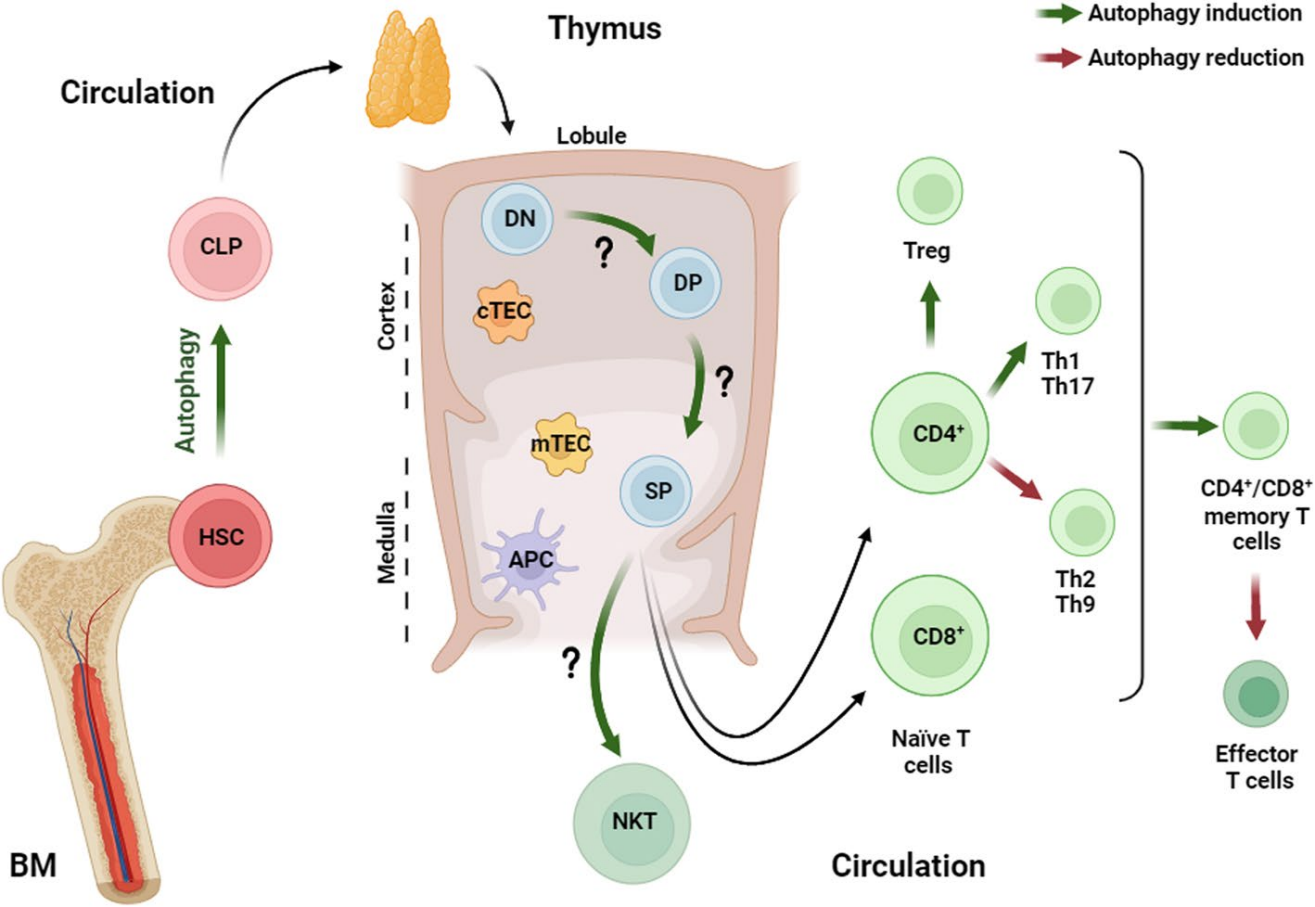
Autophagy and T cell development. Hematopoietic stem cells (HSCs) arise in the bone marrow (BM) and migrate into the peripheral blood circulation as common lymphoid progenitors (CLP), finally reaching the thymus tissue. In the cortical section of the thymic lobule, double negative (DN) thymocytes lacking CD4 and CD8 develop into double positive (DP) cells. By interaction with peptide-loaded MHC (pMHC) complexes of cortical thymic epithelial cells (cTECs) ensuring the functionality upon MHC stimulation, thymocytes are positively selected. In the medullary part of the lobule, thymocytes are negatively selected by the interaction with pMHC ligands on medullary thymic epithelial cells (mTEC) and antigen-presenting cells (APC) to filter out and exclude self-reactive T-cells. After selection, thymocytes undergo commitment to the CD4, CD8, or NK-T lineage to single positive (SP) cells and are released into the bloodstream as naïve T cells. Depending on interaction with class II MHC complexes and cytokines milieu, CD4^+^ cells differentiate into further subsets. The figure is created with “BioRender.com”

T cells are a heterogenous lymphocyte population extremely important in our immune system. They are characterized by two main subgroups based on their T cell receptor (TCR): αβ- and γδ-TCR. The first plays a central role in the adaptive immune response, while the second flanks NK cells as components of the innate immune system. Both T cell subsets help our body to defend against external pathogens, prevent cancer development and fight malignant cells once the immune surveillance is ineffective [8]. Differently from NK cells and in order to generate mature T lymphocytes, lymphoid progenitors, which have developed from hematopoietic stem cells in the bone marrow (BM), migrate to the thymus to complete their antigen-independent maturation. Inside the thymic microenvironment, they undergo thymic education through positive and negative selection acquiring important T cell markers including TCR, CD3, CD4 or CD8, and CD2. Briefly, when lymphoid progenitors arrive in the thymus, they express neither CD4 or CD8 and are therefore classed as double-negative (DN) (CD4^−^CD8^−^) cells. Then, they start to interact with thymic elements triggering the maturation process becoming double-positive thymocytes (CD4^+^CD8^+^) and finally maturing into single-positive (CD4^+^CD8^−^ or CD4^−^CD8^+^) populations that are released from the thymus to peripheral tissues. Typically, these mature thymocytes are still referred to as “naïve population” since they have not been presented with an antigen. Lastly, they travel to secondary lymphoid tissue, such as the lymph nodes and tonsils, where antigen presentation will occur facilitating the development of antigen-specific adaptive immunity (Fig. 2).

### Autophagy in hematopoietic stem cells (HSCs)

Several studies revealed that HSCs are characterized by both high glycolytic activity and high levels of basal autophagy which demonstrated to be crucial for these long-living quiescent cells [9, 10]. Accordingly, Mortensen et al*.* proved that, when *Atg7* is downregulated in vivo*,* the accumulation of Reactive Oxygen Species (ROS) damages mitochondria and DNA, impairing the maturation of HSCs into myeloid and lymphoid progenitors [11]. They also reported that loss of autophagy leads to an abnormal proliferation of the myeloid compartment with the occurrence of myeloproliferative disorders/neoplasms in vivo [12]. Furthermore, Warr et al*.* demonstrated that the survival of HSC populations facing metabolic stress depends on autophagy and is mediated by the autophagy-inducing transcription factor Forkhead Box (Fox) O3A (FoxO3A) [13]. Lastly, selective CMA is important for protein quality control and metabolic adaptation following HSC activation [14], underlining the significance of autophagy for maintaining differentiation capability of HSCs.

### Autophagy in thymocytes and during T cell development

Autophagy plays an important role in shaping T cell repertoire and developing central tolerance. The basic principle of both T cell development and repertoire formation relies on the unique role of the thymus. Several million T cells are produced every day, but only a small subset “passes the quality check” and is released into the bloodstream. The quality check consists of two selection mechanisms mediated by the interaction among premature T cells, thymocytes and epithelial cells in the thymus. T cell recognition is based on the interaction of the TCR and peptide-loaded major histocompatibility complexes (pMHC) on the surface of antigen-presenting cells (APC). The peptide specificity of the TCR is emerging through random rearrangement of the variable (V), diversity (D) and joining (J) segments of the TCR loci. This process leads to a high TCR diversity, including non-active or hyperactive T cell specificities. Non-active species are unable to be stimulated via MHC, while the others strongly react to endogenous epitopes, leading to autoimmunity. In particular, during thymic T cell maturation, CD4^−^CD8^−^ cells proliferate via Wnt signaling and differentiate into CD4^+^CD8^+^ population. When this population enters into the thymus, they migrate through the cortex and interact with pMHC ligands on cortical thymic epithelial cells (cTECs). The first selection ensures that T cells become able to react to a *stimulus* (“positive selection”) and also determines the commitment to CD4 or CD8 lineage respectively. Here, double-positive T cells expressing αβ-TCR develop to CD4^+^ T cells recognizing MHC-II bound molecules while CD8^+^ T cells remain able to bind MHC-I restricted peptides. Upon positive selection, both CD4 and CD8 cells receive a survival signal and subsequently move further to the medullary part of the thymus. Here, they encounter pMHC ligands on medullary TECs (mTECs) and dendritic cells (DCs). TCR engagement at this stage triggers an apoptotic suicide program. Therefore, the second stimulus excludes all T cells, which are reactive to self-antigens (“negative selection”). In this scenario, Klein and colleagues found that autophagy supports CD4^+^ T cell tolerance by facilitating the direct presentation of endogenous self-antigens by mTECs [15]. It was shown that TEC cells express higher constitutive basal autophagy activity compared with other tissues (60% in cTEC and 10% in mTEC) [16, 17]. Nedjic J. et al*.* investigated the role of Atg5 on T-cell selection showing that *Atg5* knockout in mice led to an only altered selection of certain MHC-II class restricted TCRs [17]. In fact, the authors demonstrated that *Atg5*^–/–^ mice developed a severe wasting disease, with CD4^+^ T cell inflammatory infiltrates in several organs. This evidence supports the idea that constitutive autophagy expression in TECs is involved in generating T cell repertoire.

## Autophagy in regulatory T cells (Treg)

Treg cells rely on Fatty Acid Oxidation (FAO) and oxidative phosphorylation (OXPHOS) for providing immune regulation in low-glucose, lactate-rich environments such as the intestinal tract [18]. Several studies report that Treg differentiation, survival and activity are associated with increased autophagic activity (Fig. 2). Wei et al*.* showed that Treg-specific deletion of both *Atg5* or *Atg7* lead to the loss of this population by favoring both mTOR and c-myc pathways and up-regulating glycolysis, causing a metabolic shift unfavorable for Treg function and survival [19]. Interestingly, the pro-autophagy protein AMBRA1 (autophagy and beclin 1 regulator 1) has been reported as a positive regulator of transcription factor FOXP3 [20]. FOXP3 serves as a lineage specification factor of Treg cells, regulating Treg development and functions. In details, AMBRA1 is a positive regulator of the BECLIN 1-dependent programme of autophagy and, through its ability to bind the protein phosphatase 2A (PP2A), stabilizes FOXO3, triggering FOXP3-mediated transcription, and T cell differentiation and homeostasis.

Considering the interplay between autophagy and Treg generation and maintenance, autophagy could play a significant role in the pathophysiology of various autoimmune diseases. Parekh et al*.,* indeed, showed that *Vps34* deficiency in T cell population reduces differentiation of Treg cells leading to inflammatory wasting syndrome in mice [21]. Moreover, Wei et al. associated knockout of both *Atg5* and *Atg7* with autoimmune inflammatory disease triggered by impaired Treg differentiation [19]. In line with these results, deletion of *Atg16l1* impaired the induction of FAO pathway genes and loss of Treg in the intestine, resulting in a more severe form of inflammatory bowel disease with an imbalance between Th2 and Treg populations [22]. Autophagic flux was also diminished in Treg cells in a model of systemic lupus erythematosus with IL-21 inhibiting their differentiation by stimulating mTORC1 and mTORC2, whereas treatment with rapamycin could restore autophagy and normalize Treg function [23]. Notably, IL-21 also drives deficiency of Treg cells via upregulation of pyroptosis in an Akt-mTOR-dependent manner in nasal polyps of patients with eosinophilic chronic rhinosinusitis, as stated by Chang et al*.* [24]. Interestingly, reduced autophagy has been found to increase reactive oxygen species to activate NLRP3–caspase 1–induced pyroptosis [25]. Furthermore, Treg plays a role during hepatitis B virus infection by preventing immune-mediated liver damage [26]. In that regard, Cheng et al*.* showed the importance of high mobility group box 1 (HMGB1)-induced autophagy for maintaining Treg function during infection [27]. In contrast, another study showed that autophagy inhibition restored Treg/Th17 balance in an in vivo model of allergic rhinitis [28]. Altogether, these studies underline the importance of a fine-tuned autophagic flux for maintaining Treg cell population regulating immune-balance.

## Autophagy in CD4^+^ T helper cells

Autophagy has been found to be required for the generation of effector CD4^+^ T cells from naïve T cell population as demonstrated in a murine model with *Beclin1 *T cell-specific deletion [29]. Importantly, multiple studies suggest that the role of autophagy differs among CD4^+^ T cell subsets (Fig. 2). Whereas blockage of autophagy favors Th2 and Th9 sub-populations, it commonly proves a disadvantage to Th1 and Th17 compartments. Kovacs et al*.* reported that the deletion of *Beclin1* in T cells results in accumulation of apoptotic proteins causing both Th1 and Th17 cell death [30]. Similarly, autophagy inhibition after *Atg16l1* deletion enhanced Th2 survival in murine intestinal mucosa while reducing Th1 and Treg populations [22]. Furthermore, knockout of both *Atg3* and *Atg5* resulted in increased IL-9 production in Th9 cells leading to improved tumor control, whereas autophagy activation suppresses their differentiation by selectively degrading Th9 cell transcription factor PU.1 [31, 32]. Lastly, Robins and colleagues defined a CD8^+^ MHC-II-recognizing population derived from effector CD4^+^ T cells and demonstrated that deletion of both *Vps34* or *Atg7* favors their generation, making autophagy an important regulator of differentiation even across the main T cell subsets [33].

Interestingly, it has been reported that *Pik3c3*-deficient CD4^+^ T cells fail to differentiate into Th1 cells associated with lower interferon γ (IFNγ) expression and a decrease in active mitochondria upon activation. Hereby, *Pik3c3*^*f/f*^*;CD4-Cre* mice proved to be resistant to experimental autoimmune encephalomyelitis, highlighting the possibility to target CD4^+^ T cell differentiation for the treatment of inflammatory diseases [34]. Cen et al*.* were also able to demonstrate that regulation of autophagy in mesenchymal stem cells directly affects CD4^+^ T cell differentiation through C-X-C motif chemokine ligand (CXCL) 8 and Tumor Growth Factor (TGF) β1. Contrary to mentioned reports, upregulation of autophagy by rapamycin was found to increase the *ratio* of Treg, whereas repression via 3-methyladenine supported the differentiation into Th1 cells [35]. In line with this, Amersfoort et al*.* observed that *Atg7* knockout in a murine model of diet-induced steatosis favored Th1 and Th17 differentiation by increasing IFNγ and IL-17 expression in both CD4^+^ and CD8^+^ T cells whilst resulting in decreased inflammatory potency and reduction of experimental atherosclerosis [36]. Using an asthma murine model, Zhao et al*.* reported, instead, that *Atg5* knockout led to a reduction of both Th1 and Treg populations and an increase of Th17 subpopulation counteracting immune imbalance, highlighting autophagy-inhibition as a possible mechanism underlying acupuncture treatment for asthma [37].

Although these last two studies seem contradictory, a more in-depth analysis of Amersfoort's study reveals that, in the livers of *Atg7*-deficienc mice, the absolute number of both CD4 and CD8 T cells was significantly reduced compared to the control group; therefore, the total amount of IFNγ and IL-17 produced by T cells throughout the process of hepatic steatosis development was significantly lower in *Atg7*-deficient mice. Furthermore, in the same study, using 3-methyladenine or ammonium chloride and leupeptin, the authors demonstrated that autophagy inhibition in Th1 cells was able to impair IFNγ secretion, suggesting that it is unlikely that *Atg7* deficiency is able to increase Th1 differentiation resulting in enhanced IFNγ secretion.

## Autophagy in CD4^+^ and CD8^+^ memory T cell formation

In CD8^+^ T cells and later also confirmed in CD4^+^ T cells, autophagy not only promotes cell survival [38] but, most importantly, enables the establishment of memory populations (Fig. 2). Notably, the maturation from naïve to memory induces a metabolic change in the cells in order to meet their new physiological needs. While CD4^+^ Th and CD8^+^ effector T cells rely mostly on glycolysis for energy generation, CD4^+^ Treg and CD8^+^ memory T cells revert to OXPHOS, in particular lipid oxidation [9]. This metabolic switch and the upregulation of autophagy during memory transition share the same regulatory machinery, underlining a strong reciprocal influence. AMPK signaling and inhibition of mTOR promote the transition from effector to memory phenotype and are, at the same time, an integral part during autophagy activation [39].

Furthermore, it has been reported that when autophagy is blocked by *Atg5* or *Atg7* deletion, CD8^+^ T cells conserved physiological function and proliferation, but impaired capacity to survive and become memory T cells [40]. Similarly, Puleston et al*.* demonstrated that *Atg7*^*−/−*^ murine T cells were not able to establish a CD8 memory compartment in vivo in response to both influenza and cytomegalovirus infections [41]. Following studies revealed that this phenomenon is not limited to the CD8 population but is also observable in CD4^+^ T cells [38].

Intriguingly, there are controversial reports on the consequence of autophagy-dependent memory formation in the tumoral context. DeVorkin et al*.* showed that inhibition of autophagy aids the formation of CD8^+^ effector memory population and *Atg5*-deficient CD8^+^ T cells increased IFNγ and Tumor Necrosis Factor α (TNFα) production, achieving better tumor control [42]. Contrarily, Vodnala et al*.* reported that, upon starvation conditions, CD8^+^ T cells undergo changes in histone acetylation showing less exhaustion and leading to an improved tumor control [43]. Nevertheless, the exact pathways through which autophagy affects the metabolism and epigenetics of T cells, modulating their differentiation and function in a cancer context, remain to be further elucidated.

## Autophagy upon T cell activation

**Fig. 3:**
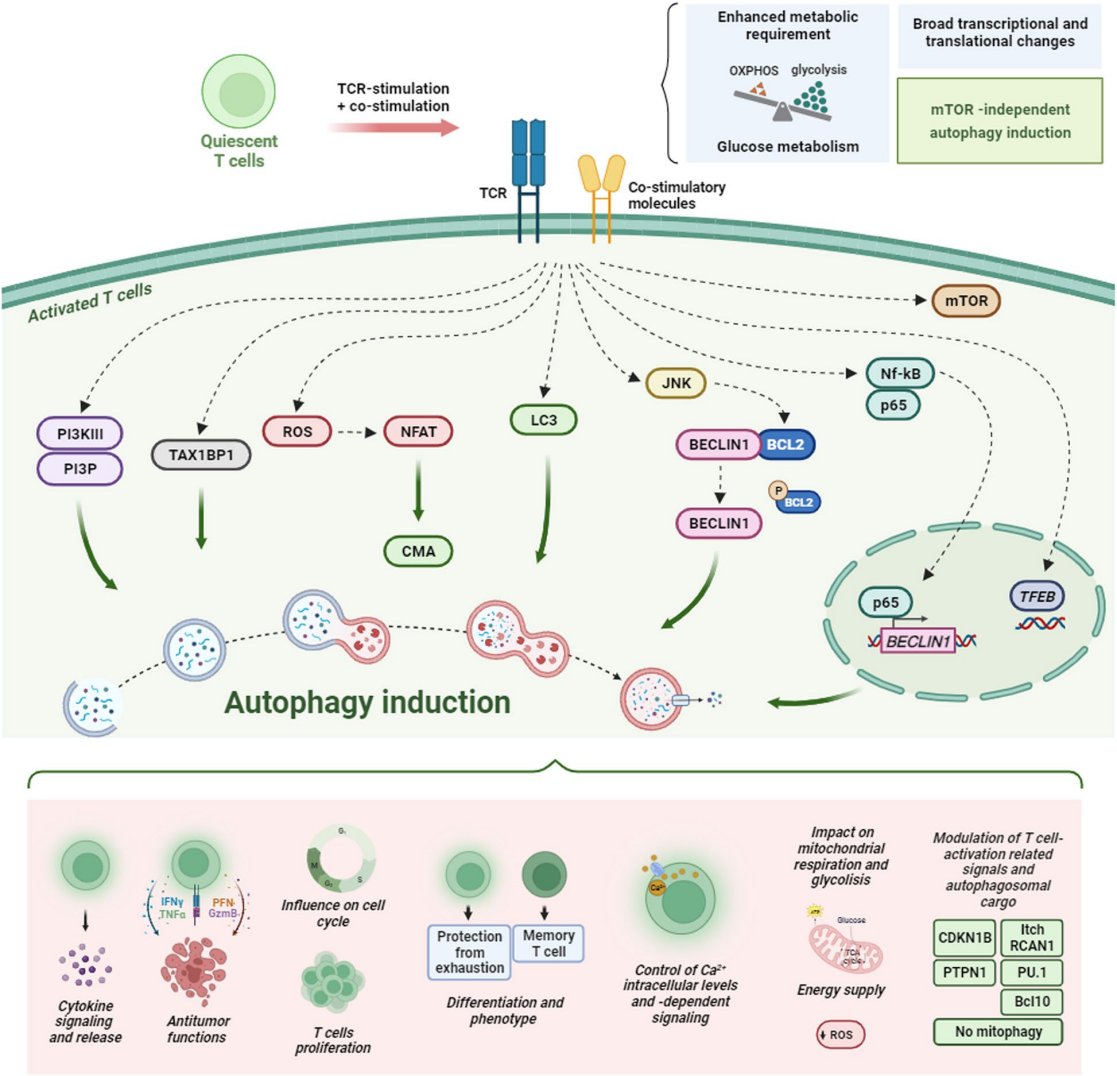
Autophagy and T cell activation. When T cells are activated by TCR-stimulation and co-stimulation, these cells undergo a metabolic shift from oxidative phosphorylation (OXPHOS) to glycolysis to meet the new enhanced metabolic requirements. Furthermore, this activation induces important transcriptional and translational changes mainly driven by mTOR-independent autophagy induction capable to activate and modulate the autophagy machinery. Activation-induced autophagy then contributes to: modulation of cytokine signalling and release; mediation of antioxidant effects; promotion of T cell survival and proliferation as well as sufficient ATP production and energy supply; control of intracellular calcium levels and calcium-dependent signalling; active impact on T cell fate by determining T cell differentiation and phenotype; modulation of mitochondrial respiration and glycolysis; influencing T cell function; impact on T cell cell-cycle control; modulation of cellular levels of specific T cell activation-related signalling players/autophagosomal cargo (Cyclin-dependent kinase inhibitor 1B (CDKN1B), itchy E3 ubiquitin protein ligase (Itch) and Regulator Of Calcineurin 1 (RCAN1), Interleukin-7 receptor subunit alpha (Il-7Rα), transcription factor PU.1, Protein Tyrosine Phosphatase Non-Receptor Type 1 (PTPN1), B-cell lymphoma/leukaemia 10 (Bcl-10), “selective exclusion” of mitochondria from autophagosomes). The figure is created with “BioRender.com”

As described previously, quiescent T cells mainly rely on OXPHOS under oxygen-rich conditions, while newly activated T cells undergo a switch to the glycolytic pathway. Accordingly, the transition to activated effector T cells represents a bio-energetically challenging process with broad transcriptional and translational changes [44]. Upon activation, T cells upregulate both glucose metabolism and autophagy to ensure adequate energy supply (Fig. 3) [44].

It is generally accepted that activation of T cells requires not only stimulation of the TCR by peptide presentation through an MHC complex, but also co-stimulation (e.g. by CD28), for complete activation. This double stimulus triggers transcriptional changes, including the nuclear factor-κB (NF-κB), activator protein 1 (AP-1), nuclear factor of activated T cells (NFAT) and induces the activation of phosphoinositide 3-kinase (PI3K) and mTOR promoting T cell growth, proliferation and survival [45, 46].

Previous studies have shown that macroautophagy is induced in CD4^+^ and CD8^+^ T cells in response to TCR engagement [47–49] regulating the cellular levels of specific signaling molecules and modulating T cell metabolism (Fig. 3) [50]. Hubbard and colleagues also reported that one of the main roles of macroautophagy in the context of T cell activation is providing an adequate energy supply to determine T cells' fate to be activated or to become anergic [48].

Interestingly, a concomitant activation of autophagy and mTOR signaling in response to TCR engagement was reported in several studies for CD4^+^ and CD8^+^ T cells, in which the inhibition of mTOR did not or only slightly induce autophagy, indicating a mTOR-independent mechanism of autophagy regulation in T cell activation [47, 51]. Aiming to exclude a mechanism of initial mTOR activation and later mTOR inhibition, Botbol et al. conducted an experiment monitoring the phosphorylation status of RPS6KB (p70)/p70-S6K (ribosomal protein S6 kinase B 70 kDa) protein, a mTOR substrate, under rapamycin addition at different time points, confirming maintained mTOR activity [51]. As a possible alternative, a pathway has been proposed in which Jun kinase (JNK) phosphorylates B-cell lymphoma 2 protein (Bcl-2), causing its dissociation from Beclin1, resulting in the induction of autophagy, as previously shown in other settings [50, 52]. Indeed, in murine CD4^+^ T cells, it has been demonstrated that Jnk-dependent pathways are required for a potent induction of autophagy [49]. Furthermore, the same group confirmed the importance of the class III PI3K complex for induction of autophagy [49]. Conversely, it has been shown that T cells with defects in the class III PI3K complex show deficits in the appropriate response to TCR stimulation [21, 53]. Another point supporting the concept of a specific, mTOR-independent pathway of autophagy activation after TCR engagement is provided by a study by Whang et al*.,* showing that Tax1-binding protein 1 (TAX1BP1), an autophagy receptor, specifically drives autophagy in early stages of T cell activation to provide L-cysteine and other amino acids which contribute to the activation of mTOR complexes and subsequent mTOR-dependent biosynthetic and bioenergetic transformations, thus driving changes required during T cell activation [54]. Importantly, global autophagy is not impaired in *Tax1bp*^*−/−*^ T cells, thus underlining its specific function during TCR-activated autophagy [54].

A critical factor for potent T cell responses is the number of T cells in the periphery. Jia and colleagues demonstrated that *Atg3*-deficient T cells showed a reduction in long-term survival and this effect correlated with both ER and mitochondrial expansion over time, supporting an autophagy-dependent control [55]. Similarly, in other studies also *Atg5*- and *Atg7-*deficient murine T cells exhibited a significant reduction in survival and absolute peripheral number [56, 57]. Furthermore, mice deficient for the essential autophagy genes like *Atg3, Atg5* and *Atg7* in both CD4^+^ and CD8^+^ T cells displayed defects in activation-induced proliferation [48, 53, 55, 56, 58]. Another study showed both proliferation and survival deficits in murine T cells with a deficiency in *Rab7,* essential for autophagosome degradation [59], pointing out the far-reaching importance of several players within the autophagy pathway for T cell activation. Hubbard et al*.* reported that autophagy-deficient T cells are unable to degrade Cdkn1B, one of the main negative cell-cycle regulators, after T cell activation, preventing them from entering in the S phase of the cell cycle after TCR stimulation, leading to impaired proliferation [60].

A possible mechanism for mTOR-independent autophagy activation has been described in activated Jurkat cells; NF-κB family member p65, which was described to be engaged after TCR activation, mediates upregulation of Beclin 1 protein levels with subsequent induction of autophagy [61]. Additionally, Botbol et al*.* reported a post-transcriptional up-regulation of LC3 protein levels despite a concomitant enhanced LC3 turnover following the induction of autophagy after TCR engagement in CD4^+^ T cells, potentially representing the ability to rapidly further increase autophagic flux [51].

Besides that, different autophagy-dependent mechanisms have been proven to be induced after TCR activation. One example is represented by the degradation of Bcl-10, protecting the cells from excessive NF-κB activation [62]. Another example is represented by the “selective exclusion” of mitochondria and other organelles from forming autophagosomes [48]. Furthermore, Mocholi et al*.* reported that T cells with blocked autophagy failed to sufficiently execute the selective degradation of the protein tyrosine phosphatase PTPN1 in response to TCR signaling, resulting in an enhanced expression of anergy-associated genes (*EGR2, EGR3, TLE4,* and *GRAIL)* and impaired T cell function [63].

In addition to macroautophagy, CMA has been reported to be activated in T cells after TCR engagement. After T cell activation, enhanced levels of CMA-related lysosomal receptor LAMP-2A are observed, with TCR-induced ROS production and subsequent NFAT activation [7, 64]. The main role in T cell activation is fulfilled by CMA through the selective degradation of negative regulators of T cell activation, such as the ubiquitin ligase Itch and the calcineurin inhibitor RCAN1. Accordingly, the deletion of *LAMP-2A* reduces activation-induced proliferation and cytokine secretion as well as impaired infection defense and immunization response [51, 64]. Furthermore, transcription factor EB (TFEB), known as a master transcriptional regulator of lysosomal biogenesis and autophagy, was reported to be upregulated in response to T cell activation and directly linked to lysosomal numbers within the cytoplasm, suggesting TFEB as the direct link between the lysosomal pool and CMA [64].

In contrast to previously described findings, Xu et al*.* observed a decrease in autophagic flux in virus-specific CD8^+^ T cells during the clonal expansion phase and an increase in autophagy at the peak of T cell expansion just before the entry into contraction phase with subsequent memory formation [40]. Furthermore, serious defects in memory formation with concomitant increased cell death, impaired mitochondrial fatty acid oxidation, and increased GLUT1 expression were reported in both *Atg5* or *Atg7*-deficient CD8^+^ T cells [40, 41]. In contrast, another study on murine *Atg5*^*−/−*^ T cells demonstrated a significant reduction in effector CD8^+^ T cell proliferation in response to viral infection [65]. In contrast, DeVorkin et al*.* described that *Atg5* deficiency in T cells promoted the generation of CD8^+^ effector memory cells, glycolytic metabolism, mediating changes in histone methylation influencing T cell activation and metabolism and enhancing antitumor activity [42].

Aiming to exploit strategies to improve T cell antitumor activity by enhancing autophagy, Chakraborty et al*.* found that a slight increase in ER stress induced by carbon monoxide (CO) activated ER sensor protein kinase R-like endoplasmic reticulum kinase (PERK) which induced autophagy and mitochondrial function, led to epigenetic reprogramming and significantly increased antitumor T-cell function in vivo [66].

Another mechanism described that elevated extracellular potassium levels—a condition found within the immunosuppressive microenvironment of solid tumors due to cell necrosis—led to decreased consumption of extracellular energy sources, creating a state of functional starvation with concomitant induction of autophagy and mitochondrially driven energy production [43].

Interestingly, Guerrero-Ros et al*.* published a study demonstrating impaired human CD4^+^ T cell responses through impaired autophagy due to increased lipid load [67], which highlights the transferable relevance of impaired T cell activation and function due to impaired autophagy caused by external factors for clinical research.

Taken together these studies highlight that autophagy plays a crucial role in T cell activation and function and its impairment causes drastic restrictions in functional T cell response (Fig. 3). However, the exact mechanisms and alternative pathways involved in T cell activation-dependent autophagy require further investigation.

## Autophagy and generation of energy upon T cell activation

During macroautophagy, autophagic cargo within the cytoplasm is getting engulfed by an isolated membrane forming the autophagosome which later will fuse with a lysosome in order to degrade and recycle the cargo [68]. Interestingly, the content of autophagosomes in T cells has been shown to change significantly after their activation, reducing the number of cellular organelles and mitochondria and increasing the amount of soluble cytosolic components [48]. In addition, reduced utilization of fatty acids as a result of autophagy blockade was observed and associated with autophagy, which may specifically indicate reduced utilization of lipophagy for energy production [48].

A possible explanation for this observation is based on the fact that the activation of T cells results in a higher ATP demand and that mitochondria play a major role in intracellular calcium signaling so that other cytosolic molecules are degraded in order to cover the energy request [48, 69]; in this scenario, macroautophagy demonstrated to play a central role in energy homeostasis [48, 63]. To analyze the effect of autophagy on mitochondrial respiration and aerobic glycolysis in TCR-activated T cells, Yang et al*.* found a drastic reduction in oxygen consumption rate (OCR) and extracellular acidification rate (ECAR) in *Pik3c3*-deficient CD4^+^ and CD8^+^ T cells, suggesting widespread negative impairment of both metabolic pathways when autophagy is defected [34]. These findings in autophagy-deficient T cells are in line with the observation of reduced OCR and ECAR after T cell activation when autophagy is inhibited [63]. Interestingly, the exogenous addition of IL-2 to the autophagy-blocked T cells caused the restoration of OCR in response to TCR engagement [63].

## Relationship between cytokine release and autophagy in T cells

Besides TCR engagement and co-stimulation, cytokine signaling and release fulfill a major role in T cell function, metabolism and activation [70]. Most cytokines play a critical role in the modulation of immune cell activation, proliferation and differentiation [71].

Autophagy was shown to be selectively induced in T cells after stimulation by common γ-chain family cytokines, like IL-2, IL-4, IL-7 and IL-15 [49, 51, 72]. For example, Ara et al*.* described the effects of the pro-survival cytokine IL-15 on T cells, activating AMPKα1 and upregulating ULK1, ATG7, the mitochondrial fusion protein optic atrophy-1 (OPA1), TFAM, AQP9, CPT1α and Complex I mitochondrial biogenesis protein levels respectively, while also leading to reliance on FAO with concomitant downregulation of hypoxia-inducible factor (HIF)-1α and thus glycolysis [73]. Interestingly, this mechanism involving γ-chain family cytokines was also shown to be crucial for the complete induction of autophagy in response to TCR and CD28 engagement. This also reiterates the important role in activated T cells of both autocrine or paracrine loops for autophagy induction after the activation-dependent cytokine release [51].

Knowing that autophagy is required for CD8^+^ memory generation and that common γ-chain cytokines appear to induce autophagy, Botbol et al*.* speculated whether cytokine-induced autophagy might be important for memory generation [40, 51].

In addition to the cytokines’ mediated autophagy-activating functions, cytokines were also being reported to induce autophagy inhibition like the immunosuppressive cytokine IL-35, which is able to impair CD4^+^ T cell proliferation and differentiation in sepsis, presumably by reducing HMGB1-dependent autophagy pathway [74].

Regarding the effects of T cell autophagy on cytokine production and secretion, it is important to underline that autophagy blockage by *Atg7* knockout in murine effector T cells, has been shown to cause notable defects in IL-2 and IFNγ secretion, in line with the above-mentioned observation of impaired T cell activation and proliferation after TCR stimulation in case of autophagy blocks [48]. Then, this data was confirmed by another study where the authors demonstrated that T cells, activated in the presence of autophagy inhibitors, showed a decrease in IL-2 production, a phenomenon that could be partially reversed by an exogenous ATP administration [63]. In contrast, in *Atg5-*deficient CD8^+^ T cells the production of IFNγ and TNFα in-vitro and IFNγ *in vivo* was increased [42]. This data was then confirmed in other two studies where *Atg7*-deficient CD4^+^ T cells, both after CD3 or CD3/CD28 stimulation, and *Atg3-*deficient T cells proved to produce elevated levels of IL-2 [55, 62]. Like macroautophagy, also CMA plays a role in the production and secretion of cytokines: CMA impairment, indeed, after *Lamp-2a* depletion in CD4^+^ T cells resulted in significantly lower secretion of both IL-2 and IFNγ [64].

Furthermore, it is important to mention that in various animal experiments, the blockade of autophagy led to an excessive, mainly cytokine-induced inflammation and a significant reduction of peripheral T cells [21, 22].

As an example, Th9 cell master transcription factor PU.1, usually degraded by p62-dependent autophagy machinery, has been reported to mediate an increase of IL-9 secretion, which has important relevance in the anticancer activity [32].

Recently, Chao et al*.* reported that under glucose deficiency-a condition typical for the tumor microenvironment (TME)– autophagy-dependent glutaminolysis was involved in the feasibility of the required IFNγ production of CD8^+^ T cells [75].

Apart from the above-mentioned, the multifaceted interactions between autophagy, cytokines and the adaptive immune response, which seem to differ between the different cell types in a relevant manner [51] and thus require further investigation, have been reviewed in detail elsewhere [70, 71, 76–78].

## Autophagy and T cell homeostasis

Several studies in autophagy-compromised genetic models have clearly revealed the critical role of autophagy in keeping T cells in homeostasis while supporting the adaptation to changes within their environment [50, 53, 55, 56]. As described for other cellular subsets, autophagy is induced in T cells under starvation conditions in order to provide building blocks needed for the synthesis of new cellular components and the maintenance of cellular energy levels [7, 49]. Additionally, as mentioned so far, several mechanistic studies illustrated how autophagy is crucial for keeping the organelles and other cytoplasmic components in balance within the cytoplasm and acting as a general quality control mechanism [50].

### Impact of mitophagy on T cell survival and homeostasis

One of the main factors for the maintenance of T cell homeostasis is represented by mitophagy [79–81]. As previously described, during the transition from thymocytes to mature T cells, a change in mitochondrial volume can be observed. Pua et al*.* found a drastic reduction in *Atg*7-deficient mature murine T cells in the spleen and lymph nodes with concomitant distinct increase in apoptotic cell death cells, while numbers of thymocytes were only borderline decreased. Furthermore, in mature *Atg7*-deficient T cells, an approximately twofold increase in mitochondrial content was detected, which was accompanied by an approximately twofold increase in ROS, a marked disturbance in the balance of pro- and anti-apoptotic factors—mainly represented by an increase in Bak—and an increase in the death-inducing mitochondrial protein cytochrome c and apoptosis inducing factor (AIF). Importantly, this phenomenon seems to be more pronounced in CD8^+^ T cell than in CD4^+^ compartment. These observations were then further corroborated by other studies monitoring CD4^+^ and/or CD8^+^ T cells. In these studies, an increase of cell death, impaired homeostasis and mitochondria accumulation were detected when autophagy was impaired (by the deletion of *Atg3, Atg5, Atg7, Beclin1, Vsp34* genes respectively, or by pharmacological inhibitions) [21, 30, 53, 56, 58, 82, 83].

Furthermore, the role of mitophagy during T cell activation was reported also by Hubbard et al. who observed a selectivity mitochondrial exclusion during the autophagy process which might be due to mitochondrial fusion-fission events [48]. Both these two processes, fusion and fission, have been largely described to be involved in the processes of mitochondrial regulation [84]. Moreover, other studies underlined the importance of mitochondria after T cell activation, reporting a decrease of both oxygen consumption rate and extracellular acidification rate following autophagy blockage, pointing out metabolic disruptions due to autophagy impairment [48, 63]. Then, mitochondria have also been shown to play a regulatory role in the dynamic regulation of calcium-dependent signaling cascades [55, 69, 85].

Another link between the autophagy pathway and mitochondrial function is represented by TFEB which was proven to be importantly involved in transcriptional regulation of mitochondrial integrity and function genes [86]. To study the relevance of mitophagy to T cell function and phenotype, Yu et al. investigated the metabolic fitness and antitumor activity of CD8^+^ tumor-infiltrating T lymphocytes (TILs) [87]. They found that an accumulation of dysfunctional depolarized mitochondria with compromised membrane potential was due to defects in mitophagy. Then, they reported that these TILs were dysfunctional and tended toward a terminally exhausted phenotype. Finally, since nicotinamide adenine dinucleotide (NAD) has been reported to stimulate mitophagy, supplementation of T cells with precursors of NAD such as nicotinamide riboside (NR) was performed, demonstrating that the accumulation of depolarized mitochondria and mtROS were significantly reduced in a Dynamin-Related Protein 1 (DRP1)-dependent manner, while an improvement of antitumor effector functions was detected [87]. In line with that, a study by Vardhana et al. showed that chronic antigen stimulation in T cells led to mitochondrial dysfunction, resulting in compromised T cell proliferation, due to limited ATP production and nucleotide triphosphate synthesis, and T cell exhaustion, while redox balance-maintaining treatments were able to enhance T cell self-renewal and anti-tumor functions [82, 88]. Together these studies point out the importance of sustained mitochondrial fitness and mitochondrial metabolism to guarantee better T cell function and shape.

The importance of mitophagy for cellular fitness and function is further supported by a study by Swadling et al. showing that within the liver, autophagy, and specifically mitophagy, is required for liver-resident CD8^+^ T cells to prevent accumulation of depolarized mitochondria and maintain their effector functions [72]. In addition, evidence for the relevance of impaired mitophagy to the deterioration of T cell function with age has also been established in different studies [7, 89]. To counteract the problem of too low mitophagy and the resulting negative consequences for cell function and survival, D'Acunzo et al. developed an optogenetic bimodular system based on light-dependent recruitment of pro-autophagy protein AMBRA1 to mitochondrial surface, for which they were able to demonstrate potent induction of mitophagy and resulting mitochondrial clearance also in human T cells [90].

### Autophagy and ER in T cell survival and homeostasis

The ER represents one of the key cellular organelles for protein synthesis, folding and modification, which is closely regulated by the endoplasmic reticulum quality control (ERQC) system, autophagy and the Unfolded Protein Response (UPR) complexes. An accumulation of incorrectly folded proteins within the ER leads to the activation of the ER stress response with activation of sensor protein inositol-requiring enzyme 1α (IRE1α), PERK and activating transcription factor 6 (ATF6) signaling pathways, resulting in induction of the UPR. Autophagy is known to be induced in response to ER stress by the UPR, activating ER stress-mediated autophagy and ER-phagy [91]. While ER stress-mediated autophagy is mainly involved in the degradation of worn-out proteins, protein aggregates and damaged organelles, ER-phagy selectively includes and degrades ER membranes.

Focusing on ER-related autophagy in T cells, important functions during the development and selection process from thymocytes to T cells have been reported and previously described. Addressing its relevance for diseases, Lee et al. found that T cells from patients with systemic lupus erythematosus show reduced autophagy activity and increased apoptosis—in response to ER stress compared to healthy controls [92].

Although the authors were not able to demonstrate a direct link with autophagy, a relation between ATF4 expression and the Th1 cell differentiation has been reported in CD4^+^ T cells [93]. The same applies to the relationship between the IRE1α-XBP-1 pathway T cell activation and Th cell differentiation [94–97]. Also, in this case no direct relationship with autophagy has yet been demonstrated.

Similarly, a relationship between *XBP-1* expression and T cell differentiation, effector function and exhaustion has been described for CD8^+^ T cells [98, 99], but as reported for the CD4^+^ T cells, no relationships to autophagy have been proven. Furthermore, *CHOP,* which has been shown to be involved in the UPR-dependent autophagy activation, was reported to be upregulated in tumor-infiltrating CD8^+^ T cells and correlated with decreased clinical outcome in ovarian cancer patients [100], however, its connection with autophagy has not been investigated yet. As previously described, CD8^+^ T cells are able to induce autophagy after activation during their contraction phase in order to build a memory population [40]. Particularly in the context of protein accumulation and subsequent increase in ER load, Jiang et al. speculated that ER-related autophagy may play an important role in this process, but further investigations are still required [95].

Aiming to better understand the role of autophagy in ER homeostasis in T cells, Jia et al*.* found an expansion in ER content due to dysregulated autophagy, ER turnover with concomitant increased ER calcium stores and induction of the ER stress response in Atg7-deficient CD4^+^ and CD8^+^ T cells [55]. Calcium influx is a vital component of TCR signaling with the major proportion of calcium being supplied by influx from extracellular after consumption of ER calcium stocks. Alteration of ER calcium homeostasis due to an autophagic defect was shown to lead to an insufficient calcium influx from the extracellular environment, impairing T cell responses and TCR activation [55, 101].

Interestingly, by generating transient ER stress in T cells, Chakraborty et al*.* showed an increased mitochondrial fitness, biogenesis and function via PERK and subsequent induction of cytoprotective effects of autophagy. This phenomenon may be related to an increased T cell effector function and antitumor activity, which in general reveals a link between mitochondrial fitness and ER stress-induced autophagy [66].

A well-known feature in solid TME is the deficiency of L-arginine (L-Arg) [102]: García-Navas et al*.* have shown that the deficiency of L-Arg leads to ER stress and autophagy activation, which has been reported to protect T cells from apoptosis otherwise caused by L-Arg deficiency [103].

Although many mechanisms remain to be further deciphered in detail, it can be concluded that autophagy significantly influences ER homeostasis and that ER-related autophagy probably also substantially influences processes such as T cell activation, differentiation, effector function and exhaustion.

## Impact of autophagy on T cells survival and cell death

While mostly described as a cell survival mechanism, under particular circumstances, autophagy can lead to cell death. Regulation of cell deaths plays an essential role in maintaining tissue homeostasis and remodeling. Several programmed cell death (PCD) mechanisms have been recently identified including apoptosis, necroptosis, pyroptosis and autophagy-dependent cell death (ADCD) [104, 105]. In T cells, the role of autophagy has not yet been well defined, as it has been shown to be capable of promoting or inhibiting programmed cell death regulating T cell survival [30, 49].

In fact, studies where inhibition of autophagy or autophagy-related genes is able to block apoptosis and/or caspase activation, mitigating tissue damage [106]. This finding is in line with the observation that autophagy, in T cells promotes cell survival through degradation of cell death machinery-related proteins. Kovacs et al. demonstrated that Beclin1 deficient CD4^+^ T cells are susceptible to apoptosis due to the accumulation of cell-death-related proteins such as procaspase-3, procaspase-8 and BIM [30, 50]. Similar results were then obtained in another study, observing that when blocking *Beclin 1* and *Atg7* Th2^+^ cells become more resistant to cell death [49]. Both these studies revealed that the amount of accumulated specific pro-apoptotic proteins in the cytoplasm is closely regulated by macroautophagy.

## Natural killer (NK) cells: overview

NK cells are a subset of innate lymphoid cells (ILCs) that represent the first line of immunity defense against virus-infected or transformed cells [107]. Based on intrinsic abilities, they are considered a link between innate and adaptive immune systems. NK cells, indeed, can activate themselves and acquire cytotoxic modes against tumors, an ability conventionally belonging to adaptive immune cells (e.g., CD8^+^ T cells). Once activated, NK cells also exert immunomodulatory functions through the secretion of several chemokines and cytokines (IFNγ and TNFα) recruiting and activating other immune cells (B and T cells, dendritic cells, neutrophils, macrophages) [108]. NK cells primarily differentiate and maturate in the bone marrow. In particular, HSCs give rise to all blood cell progenitors, among which CLPs are precursors of all lymphocytes. Committed NK cell Precursors (NKPs), that originated from CLPs, undergo maturation to generate pre-NK cells, immature NKs (iNKs), and mature NK cells respectively driven by cytokines stimulations (e.g. IL-15) and different transcription factors. During the process of differentiation, NK cells change their phenotype to acquire cytotoxic functions. Additionally, mature NK cells typically show a characteristic immunophenotypic pattern based on the expression of CD56 and CD16 antigens. Whereas CD56^dim^/CD16^bright^ population has direct cytotoxic potential (through degranulation, Antibody-Dependent Cellular Cytotoxicity—ADCC, and cytokine production), CD56^bright^/CD16^dim^ are primarily known as immunomodulatory NK cells (through cytokines secretion) [109] and experiences conversion into CD56^dim^/CD16^bright^ active phenotype after cytokines stimulation (IL-15, IL-2) [110–112]. Cytokine stimulation is an approach to sustain NK cell activation, proliferation, and expansion in vitro. Although IL-2 and IL-15 share the same receptor subunits, in vivo stimulation of NK cells with IL-2 is reached only with pharmacological administration as IL-2 concentration required for NK cell stimulation is commonly not achieved in physiological conditions. Additionally, IL-2 in vivo administration causes some side effects, including the stimulation and expansion of Treg. Furthermore, IL-15 is capable of counteracting the inhibitory effect mediated by the tumor microenvironment. For these reasons, the most suitable cytokine to stimulate the proliferation of NK cells in vivo is represented by IL-15 [113–115].

The function capabilities of NK cells are regulated by the balance between inhibitory and activating NK receptors (NKRs) that are able to bind the corresponding ligands on tumor cells. Of interest, NK cells preferentially eliminate differentiated and stem-like tumors via ADCC or direct NK-mediated cytolysis respectively. These functions belong typically to CD56^dim^/CD16^bright^ NK cells. NK cells can also prompt extrinsic apoptosis through the interaction of Tumor Necrosis Factor-Related Apoptosis-Inducing Ligand (TRAIL) and Fas Ligand (FasL) with Death Receptors (Fas, DR4/DR5) on the cancer cell surface. This latter is instead predominantly preferred by CD56^bright^/CD16^low^ phenotype. Indirectly, NK cells contribute to tumor eradication by positively modulating immune response through cytokines and chemokines secretion to recruit and activate innate and adaptive immune cells. Hence, NK activation is mediated by both tumor priming and/or cytokine stimulation. Based on their activation status, mature NK cells can be distinguished into activated or resting phenotypes. Resting NK cells are defined as naïve NK cells not activated by cytokines (IL-2 or IL-15) or tumor priming, thus lacking markers of a recent stimulation (e.g. CD69, CD25). Hypoxia reduces the expression of NK activating receptors contributing to a resting phenotype unable to be activated after IL-2 stimulation, but maintaining CD16 expression and thus, theoretically able to mediate ADCC [116]. Although they are not exhausted NK cells, the resting phenotype is less competent in tumor eradication and contain less granzyme B and perforin granules [117–120]. Mature NK cells must be in a proliferation state to be susceptible to cancer-mediated activation. This is often dependent on the cytokines secreted by the other immune cells into TME and also the tumor by itself which negatively contributes to NK cell activation status secreting for example TGFβ or IL-10. This favors a resting phenotype, frequently found in tumor tissues in association with poor prognosis [121, 122].

## Autophagy in NK cell differentiation

**Fig. 4:**
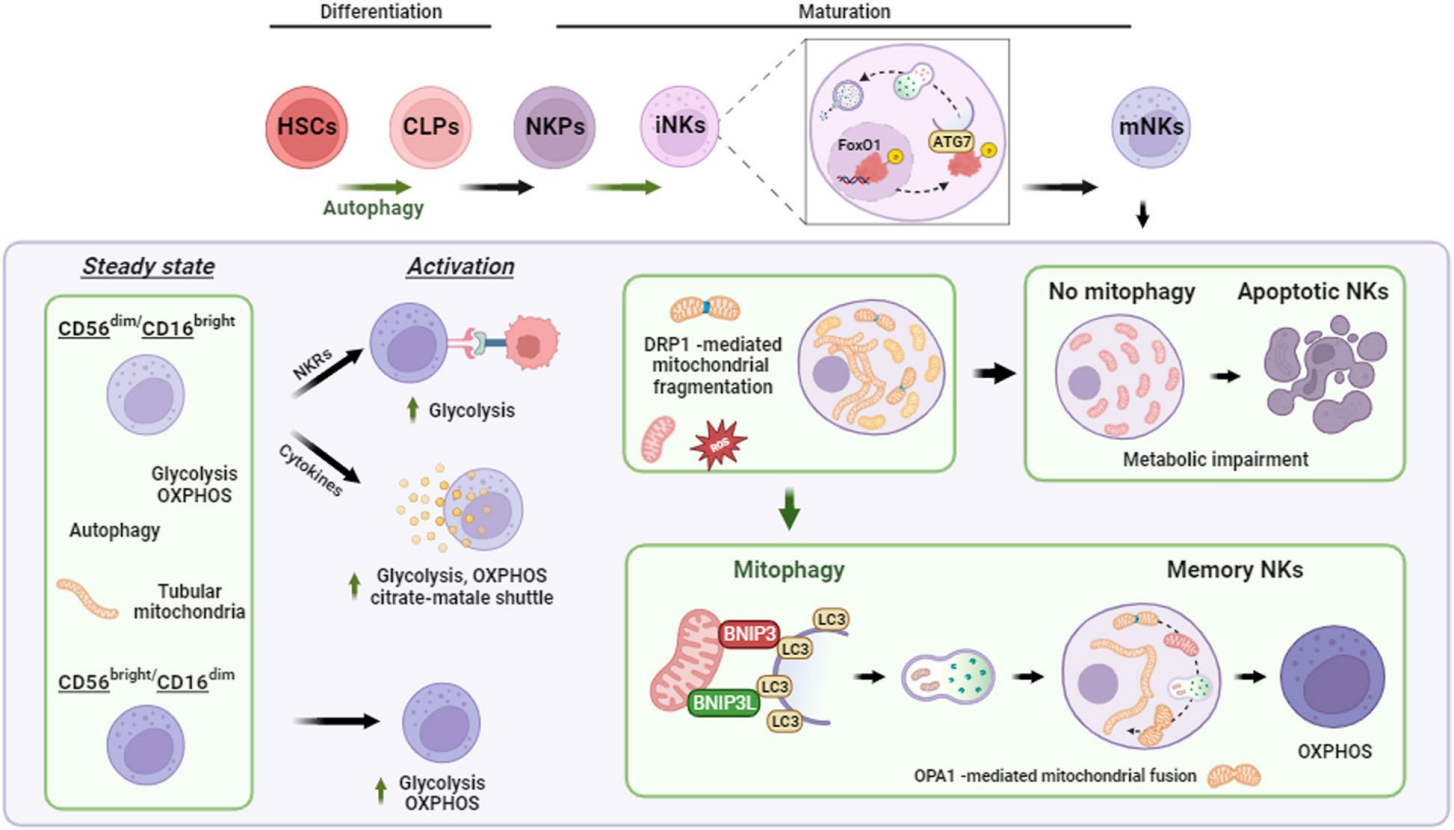
Autophagy in NK cell development and functionality. The autophagy process drives Hematopoietic Stem Cell (HSCs) differentiation into Common Lymphoid Precursors (CLP) and gains less importance in the generation of NK cell Precursors (NKP). In the stage of immature NK cells (iNK), phosphorylation of Forkhead box O (FoxO) 1 protein mediates its translocation from the nucleus to the cytosol and induces autophagy through interaction with ATG7 in the phagophore. FoxO1 is lost in mature NK cells (mNK) and autophagy is effectively reduced (green lines follow the role of autophagy in NK cell development). At steady state (freshly isolated NK cells), both phenotypes of mNK cells exhibit lower autophagy at the basal level, tubular mitochondria, and dependence on glycolysis and OXPHOS for their metabolism. After activation, the metabolism of the immunomodulatory phenotype (CD56^bright^/CD16^dim^) does not undergo metabolic reprogramming and shows greater dependence on OXPHOS than glycolysis. On the other hand, activation of cytotoxic phenotype (CD56^dim^/CD16.^bright^) induces metabolic reprogramming to fuel cytolytic activities, and glycolysis gains more importance after NK-activating Receptors (NKRs)-mediated activation, while OXPHOS and citrate-malate shuttle fuel cytokines-mediated activation. In this context, autophagy is reduced during the proliferation stage of activated NK cells, and metabolic reprogramming is supported by DRP1-mediated mitochondrial fragmentation. In the latter stages of activation, fragmented mitochondria can be recycled through BNIP3/BNIP3L-mediated mitophagy to generate memory NK cells, and OPA-1-mediates mitochondrial fusion for their biogenesis. Fragmented mitochondria alter metabolic fitness in the absence of mitophagy, leading to apoptosis of NK cells. The figure is created with “BioRender.com”

Autophagy has been shown to play a fundamental role in NK cell differentiation (Fig. 4). In NK precursors, indeed, autophagy process is essential to protect HSCs-derived iNK cells from damaged mitochondria and ROS-induced apoptosis. In particular, by generating mice lacking autophagy activity in iNKs by crossing *Atg5*^*flox/flox*^ mice with mice expressing Cre recombinase driven by the NK cell-specific NKp46 promoter (*NKp46-Cre),* the authors found that NK differentiation was blocked at the NKPs stage, thus preventing their maturation into iNK cells. Autophagy, indeed, results higher in iNKs stage than NKPs precursor or mature NK cells respectively [123]. At molecular levels, in iNKs, by means of AKT-mediated phosphorylation, the nuclear FoxO1 protein loses its role as a transcription factor and translocates to cytoplasm mediating autophagy initiation in a mTOR-independent manner. This happens through a direct interaction with Atg7 protein at the phagophore; moreover, *Atg7* expression is enhanced due to the impairment of FoxO1 transcriptional activity [123].

Metabolism and several mTOR-dependent cellular processes as well as autophagy are indispensable for NK cell development [123–125]. AKT/mTOR signaling pathway is activated during NK cell development in response to IL-15 stimulation [126]. mTOR nucleates two distinct protein complexes termed mTORC1 and mTORC2 both implicated in NK cell homing, maturation, and effector functions [127, 128]. Intriguingly, mTORCs inhibition (by silencing or rapamycin treatment) prevents NKPs progression into iNKs, while mTOR induction does not block autophagy during the NK cell differentiation process [123].

A growing number of evidence are reporting how autophagy is induced concomitantly with mTOR activation. Furthermore, several mTOR-independent autophagy pathways such as Ca2^+^, AMPK, MAPK/JNK, ROS, HIF-1α, and miRNAs have been recently identified to be able to regulate autophagy. An important question that needs to be elucidated is how autophagy avoids or counteracts the inhibitory effect of mTOR in T and NK cells.

Despite progress in this area, our understanding of this signaling network remains incomplete and many vital questions remain to be answered. However, not only mTOR kinase activity but also its subcellular localization is important for exerting its regulatory role on autophagy. Activation of mTORC1, indeed, can be heavily dependent on the lysosome that not only provides the membrane platform for the interaction of mTORC1 with its activators but is also the site where amino acids can be sensed and multiple regulatory mechanisms can be exerted [129]. This underlines how mTORC1 activity is crucial in response to nutrient oscillations and other environmental changes stimulated by feeding or fasting. Several studies underlined the role of FoxO1 as a negative regulator of NK maturation, proliferation, and activation, and loss of its expression is observed during NK cell development [130, 131]. Additionally, FoxO family proteins can induce autophagy as a consequence of different mechanisms. FoxO family proteins, indeed, regulate genes implicated in anabolic processes that are induced during nutrient depletion and negatively affect mTORC1 activity, resulting in autophagy induction [132]. Interestingly, signal transduction of NK cells activating receptor DNAX accessory molecule 1 (DNAM-1) engagement and its ligands in tumor-activated NK cells directly results in AKT-mediated phosphorylation of FoxO1 which translocates to the cytosol and undergoes ubiquitin-mediated degradation [133]. As described, cytosolic FoxO1 directly activates autophagy in iNKs but its role in activated NK cells beyond ubiquitination needs to be defined. Furthermore, although autophagy has been characterized during NK cell development, its modulation during NK education and in cancer-induced-mature NK cell activation, exhaustion or tolerance needs further investigation. Similar to CD8^+^ T cells, autophagy is reduced in the proliferation stage of antigen-specific NK cell activation upon viral infection. Accumulation of autophagosomes, indeed, was reported in expanded NK cells, supporting that autophagy is more implicated in late stages to maintain a long antiviral response [134].

### Mitochondria-related processes in NK cells

In NK cells, metabolic reprogramming influences their activation status [135, 136]. In particular, cytotoxic NK cells (CD56^dim^) maintain high levels of OXPHOS genes, mitochondrial polarization, and a high rate of both glycolysis and OXPHOS compared to CD56^bright^ cells [137]. Activated NKs require high energy demand to sustain effector functions and reprogram their metabolism based on trigger stimuli [124, 138]. Indeed, activation via IL-2/IL-15 cytokine stimulation upregulates both OXPHOS and glycolysis to mediate IFNγ release [139]. On the other hand, the glycolytic pathway seems indispensable to mediate NK cytotoxic functions in case of NK activation via NKRs engagement. Indeed, NKRs-activated NK cells increase both glycolysis and OXPHOS, but only glycolytic inhibition reduces NK degranulation, FASL expression, and hence NK killing [114].

Preserving the mitochondrial fitness of NK cells is strictly related to cellular metabolism, oxidative stress, and thus to their viability and functionality. It is interesting to note that a key role in the generation of memory NK cells has been found in the mitophagy process. Mitophagy is finely tuned and regulated through two distinct pathways: the PTEN-induced kinase 1 (PINK1)/parkin_RBR-E3_ubiquitin-protein ligase (PARKIN)-dependent and the receptor-mediated (or PINK1/PARKIN-independent) mitophagy. Of interest, when NK cells recognize virus-infected cells an increased BNIP3/BNIP3L-mediated mitophagy occurs, generating long-term memory NK cells [134, 140].

Healthy mitochondria are tubular and undergo fusion to maintain their phenotype and physiological status. During mitophagy, damaged mitochondria become fragmented (fission) and this consequent fragmentation results in ROS accumulation and metabolic impairment. If damaged mitochondria are not rapidly recycled via mitophagy, cells undergo apoptosis [141]. In liver cancer patients, tumor-infiltrating NK cells had fragmented mitochondria thus preventing NK cell activation and the consequent memory NK cell generation. Mitochondrial fragmentation alters NK metabolism through reduced OXPHOS and increases apoptosis as a consequence of hypoxic TME that induces mTOR-DRP1 signaling activation [142]. DRP1 is a major protein implicated in mitochondria fission and promotes mitochondria fragmentation. Inhibition of mitochondrial fragmentation by genetic downregulation of DRP1 or by using a fission inhibitor enhanced NK cell anti-tumor functions [142]. Additionally, mitochondrial dynamics play a crucial role in mature NK cell fitness. Comparing the impact of priming (IL-15) *versus* activation (IL-12/18) on the bioenergetics of human NK cell subsets, the authors found that CD56^Dim^CD16^+^ NK (NK^Dim^) cells amplified mitochondrial polarity upon IL-15 priming but fragmented their mitochondria after activation (IL-12/IL-18), whereas CD56^Bright^CD16^−^NK (NK^Br^) cells conserved fused and polarized mitochondria after priming and activation [137]. Interestingly, mitochondrial fusion is indispensable to maintain OXPHOS and cellular fitness in functional NK cells. Mitochondrial dynamin-like GTPase OPA1 is the main player of mitochondrial fusion and in OPA1-mutated patients, fewer CD56^dim^ cells are found and they are characterized by low mitochondrial membrane potential and altered metabolism [137]. In a recent study, Terrén and colleagues reported that in vitro stimulated-adoptive NK cells had low viability compared to unstimulated cells. Although enhanced autophagy flux was detected after cytokines stimulation, NK cell survival was closely dependent on mitochondrial clearance, and low OPA1 levels and absence of mitophagy are responsible for a consequent accumulation of dysfunctional mitochondria and NK cell death [143]. This suggests a major role of both mitochondria dynamics and mitophagy in regulating NK cell effector functions and their transition into memory cells.

Metabolic reprogramming of cancer cells is able to immunosuppress tumor-infiltrating NK cells. Warburg effect of tumor cells enriches TME of lactate and catabolites and induces TME nutrient depletion arresting NK glucose metabolism [144, 145]. Among the most important pathways implicated, autophagy proteins including ATG7 are found to increase in NK cells exposed to TME derived from ovarian cancer patients [146], suggesting that NK cells are able to positively modulate their autophagy flux in order to obtain energy and biomolecules to survive into nutrient depleted TME. Interestingly, several endogenous metabolites have been described to be able to influence mitophagy. For instance, pyruvate, a glycolytic product, enhances PINK1-mediated mitophagy, while lactate, an anaerobic glycolysis product, impairs mitophagy [147]. Hence, metabolites derived from metabolic reprogramming in activated NK cells as well as from TME could modulate mitophagy in cytolytic NK cells, thus hampering the generation of memory NK cells. Lack of mitophagy induction and an altered mitochondrial fitness in activated NK cells could contribute to cancer immune escape. Further studies should be performed to investigate and link mitophagy, mitochondrial dynamics, and metabolism in regulating intratumoral NK cell phenotype and activation.

## Autophagy in natural killer T cells (NKT)

NKT cells develop from the CD4^+^CD8^+^ population during the T cell commitment in the thymus upon TCR engagement with CD1d [148] and represent an interface between adaptive and innate immunity together with the γδ-T cells. Autophagy was shown to support the development and survival of this specific subpopulation. *Atg5* or *Atg7* conditional deletion of in the T cell compartment during their maturation impairs NKT development [36, 149, 150]. Moreover, IL-15 supports its maintenance by inducing autophagy via Tbkbp1, and its reduction leads to an overproduction of ROS and impaired NKT survival [151]. In line with that, Parekh et al*.* reported that knockout of *Vps34* in the T cell lineage impaired NKT development in the thymus [21], adding NKT cells to the list of T cell subsets depending on autophagy for differentiation. Type I NKT cells are also referred to as invariant NKT (iNKT) cells and are known to be strongly implicated in the tumor immunosurveillance as a natural adjuvant of adaptive immunity through cytokine secretion. In the latest step of tumorigenesis, iNKT cells result in hyperactivation, while other NK cells undergo exhaustion [152]. Beyond autophagy inhibition, iNKT cells also showed increased mitochondria mass and intracellular ROS [149], suggesting impaired mitophagy and increased oxidative stress. On the other hand, the deletion of the autophagy gene *Atg5* in dendritic cells increased the expression of CD1D1-glycolipid ligand stimulator complex and enhanced the activation of mature iNKT [153]. Targeting autophagy in cancer may promote tumor eradication via glycolipid processing and CD1D1 internalization resulting in increased NKT cell activation.

## Autophagy at the interface between cancer cells and NK/T cell effector functions

As reported above, autophagy plays a crucial role in both T and NK cell differentiation, activation, metabolism, and homeostasis. The interplay between tumor and immune cells composing TME is a determinant for tumor growth, maintenance, metastasis, and response to therapy. Tumor evasion from T and NK cells involves alterations in the autophagy machinery in cancer cells that negatively modulate immune cell-mediated antitumor response. In particular, high levels of autophagy in cancer cells affect several immune cells effector functions and impair their antitumor activity in different ways: modulating T and NK cell tumor recognition, degrading NK cell cytotoxic granules and influencing cytokine/chemokine secretion. Moreover, hypoxia contributes to enhancing autophagy levels in some types of tumors and promotes immune escape mechanisms [154, 155]. Understanding these mechanisms and how tumor cells exploit autophagy to regulate NK cell functions could be useful to enhance the efficacy of current treatments as well as to develop NK cell-based therapeutic approaches in combination with autophagy inhibitors.

### Tumor autophagy in T and NK cell-mediated recognition

#### MHC-I

Autophagy is upregulated in many cancer types [156] to maintain their metabolism and therefore ensure proliferation and survival [157]. Especially in solid tumors, the TME is defined by the interplay of several non-cancerous cell types and the stroma surrounding the tumor cells, providing an oxygen- and nutrition-deficient environment. These stress factors lead to switching on autophagy in order to maintain cell survival. The activation of autophagy, in turn, starts a cascade of mechanisms that lead to escape immunosurveillance and poor therapy outcomes. High mutational burden is a feature of solid tumors, which comes along with the generation of immunogenic neoantigens and the presentation to T cells via MHCI and II complexes, which, in turn allow recognition by immune cells and elimination of the cancer cells [158]. Recent studies highlight the effect of autophagy on the downregulation of MHC-I molecules and impaired neoantigen presentation, resulting in poor therapy outcomes.

LKB1 is a tumor suppressor gene and its loss of function is involved in worse overall outcomes in nonsmall cell lung cancer (NSCLC) patients. *LKB1* deficiency comes along with reduced expression of class I MHC (*HLA* genes) together with immunoproteasome activity and increased autophagic flux [159]. In a study from Deng et al. this effect was observed by inhibiting autophagy through targeting the ATG1/ULK1 pathway, leading to increased T cell infiltration and enhanced response to anti-PD1 treatment through the expansion of CD44^+^CD62L^−^ effector CD8^+^ T cells in LKB1 mutant tumors [159]. Similar observations were seen in a study by Yamamoto and colleagues, where they identified the autophagy cargo receptor NBR1, which targets MHC-I molecules for lysosomal degradation, as the cause of immune checkpoint blockade-resistant pancreatic carcinoma [160]. Intriguingly, on the other hand, enhanced MHC-I levels could hinder NK cell-mediated tumor recognition, but it cannot be excluded that other mechanisms could overcome this inhibitory signal in specific tumor settings.

In hepatocytes, Poillet-Perez et al. demonstrated that autophagy suppresses antitumor immune response through STING pathway inhibition, enhancing Treg activation and reducing IFN-γ production, thus resulting in exhausted CD8 ^+^ and CD ^+^ T cells, therefore favoring tumor growth [161]. In contrast, in an *Atg7* deficient mouse model, loss of autophagy in T cells results in greater IFN-γ production and eradication of tumors. For further confirmation of the observed effect, IFN-γ was shown to induce an upregulation of MHC I and II, therefore resulting in increased presentation of immune reactive neoantigens, which in turn lead to recognition and elimination of cancer cells [161].

#### PD-1/PD-L1 pathway

PD-1 and its ligands belong to immune checkpoint proteins implicated in the inhibition of immune-mediated response. In several tumors, PD-L1 is overexpressed on the cancer cells' surface, triggering NK and T cell desensitization and preventing their activation. Due to its relevance in immune suppression and chemotherapy resistance [162], PD-L1 and PD-1 are targeted with monoclonal antibodies, called immune checkpoint inhibitors (ICIs), and are currently approved for some advanced and/or metastatic solid tumors. Unfortunately, most patients treated with ICIs experience therapy resistance or fail to establish a long-lasting clinical response [163–165]. Also, several adverse effects as cardiotoxicity limit their applicability as anticancer agents, thus rendering it essential to identify new therapeutic strategies to enhance cancer patients’ responsiveness to ICIs-based treatment. An intriguing reciprocal relationship also exists between autophagy inhibition and PD-L1 expression on cancer cells (e.g., gastric cancer, bladder cancer, melanoma), suggesting that targeting autophagy could sensitize tumors to ICIs combined treatments [162, 166–168]. In bladder cancer cells, inhibition of autophagy is demonstrated to increase PD-L1 expression as a consequence of ERK–JNK–c‐Jun signaling pathway activation and PD-L1 negative regulator miRNA34a downregulation [167]. Then, inhibition of Vps34 kinase activity was shown to enhance PD-L1 expression in melanoma and colorectal cancers as well as PD-1 on CD45^+^ tumor-infiltrating cells (NK cells and both CD8^+^ and CD4^+^ T cells). Vps34 inhibition in combination with anti-PD-1/PD-L1 immunotherapy results in reduced tumor growth and increased survival of mice with melanoma and colorectal cancer (CRC), compared with anti-PD-1/PD-L1 monotherapy treatment [166]. Interestingly, exosomes derived by temozolomide-resistant glioblastoma (GBM) stem cells containing PD-L1 can induce autophagy with a paracrine mechanism in tumor cells via the AMPK/ULK1 mediated autophagy activation. The subsequent autophagy inhibition can overcome temozolomide resistance induced by PD-L1-containing exosomes [169]. These and other studies highlight how autophagy targeting could synergize with ICIs to overcome immunoescape mechanisms implicated in ICIs-based therapy resistance.

#### Hypoxia-induced autophagy in cytotoxic T and NK cell activity

Hypoxia is a common feature of solid tumors which modulates cell adaptation to survive in low oxygen conditions. As a result of these changes, autophagy is induced to promote tumor proliferation, therapy resistance and is essential to evade immune response [170]. The main players that drive autophagy induction in solid tumors are HIFs-α transcription factors. The cytosolic HIF-1α is normally ubiquitinated and degraded via proteasome, but in case of low oxygen environment is stabilized, translocated to the nucleus and heterodimerize with constitutively expressed HIF-1β subunit to promote autophagy through direct induction of autophagy genes expression (e.g., *BNIP3/BNIP3L*) [171, 172]. Additionally, autophagy can be induced in a hypoxic environment as a consequence of UPR activation, Signal Transducer and Activator of Transcription 3 (STAT3)/phospho-STAT3 signaling and nutrient deprivation.

In tumor cells, HIF-1α elicits an increase in pSTAT3 and autophagy pathway, promoting resistance to cytotoxic T cells (CTL)-mediated tumor cell lysis [173]. This effect was restored by blocking either *BECLIN 1* or *ATG5* with siRNA and resulted in the decrease of pSTAT3 and rehabilitation of hypoxic tumor cell susceptibility to CTL-mediated cytotoxicity [174]. There is some evidence that under hypoxic conditions, the transcription factor NANOG directly activates the expression of *BNIP3L* and contributes to autophagy in tumor cells with stem-like and immune-refractory properties which makes them resistant to CTLs [175, 176]. Direct blocking of NANOG through siRNA resulted in tumor growth inhibition and increased cell lysis through CTLs [175].

In several hypoxic tumors with high levels of autophagy, cancer cells counteract the degranulation of NK cells by regulating granzyme B degradation. For instance, clear cancer cell carcinoma (CCRC) frequently shows a mutation in Von Hippel-Lindau factor causing a constitutive stabilization of HIF-1/2α. Among HIF-2α targets, inositol 1,4,5-trisphosphate receptor type 1 directly mediates autophagy activation increasing granzyme B degradation and consequently CCRC resistance to NK cells [177, 178]. However, it is not clear how granzyme B and perforin granules enter into cancer cells. Some authors reported that NK cells release perforin that binds to tumor cell membrane, creating transient pores and Ca^2+^ cell influx to vehicle granzyme B granules into target cells by clathrin- and dynamin-dependent endocytosis. As a consequence, both perforin and granzymes are endocytosed into enlarged endosomes called 'gigantosomes' [179, 180]. On the other hand, granzyme B can enter into the cells without being enveloped in endosomes after perforin-induced pores or even in a perforin-independent manner by using other receptors (e.g. HSP70 mediated endocytosis) [179, 181–183]. In hypoxic breast and melanoma cancer cells increased autophagy caused granzyme B degradation by fusing endosomes with autophagosomes and eliminating it via autophagy machinery. Autophagy inhibition by targeting *Beclin1* or *Atg5* restored granzyme B levels in hypoxic cells in vitro and induced tumor regression in vivo facilitating NK-mediated tumor cell killing [149]. Furthermore, in NSCLC cells, autophagy inhibition with rocaglamide (a novel natural molecule capable of targeting ULK1 translation) restored tumor intracellular granzyme B, and thus susceptibility to NK cell killing [184]. Lastly, it is also important to mention that p62 targeting revealed impairment in granzyme B degradation but not perforin, therefore a speculative hypothesis suggests that granzyme B could be a selective autophagy substrate since granzyme B can enter into cells in a perforin-independent manner; further investigations are necessary to better elucidate this process. As the role of autophagy in tumor promotion or inhibition is context-dependent, also its regulation in NK cell modulation is context-dependent. Pharmacological re-activation of mutated TP53 function in breast cancer cells renders these tumors sensitive to NK cell cytolysis when autophagy is inducted in these lymphocytes. In this circumstance, restored p53 function autophagy via sestrin-AMPK-mTOR pathway and ULK axis culminates in autophagy-mediated elimination of anti-apoptotic regulators (Bcl-XL and XIAP), thus facilitating Granzyme B-induced apoptosis [185]. These findings are in line with the dual opposite effects of TP53 on autophagy regulation (both induction or inhibition based on context) [186].

Autophagy induced by hypoxic TME affects not only granzymes-mediated NK cell cytolysis but also destabilized NKs/tumor cells’ immunological synapses. Among the targets induced by HIF-1α, connexin 43 (Cx43) forms Gap-junctions to stabilize NK cells/tumor cells’ connection. Unfortunately, Cx43 shows a short half-life and its turnover depends on the balance between its HIF-1-mediated expression and its autophagy-mediated degradation [187, 188]. Autophagy induced in melanoma cells exposed to hypoxic TME alters the localization of Gap-junctional connexin 43 (GJ-Cx43), thus destabilizing immunological synapses and facilitating the escape of NK killing. Autophagy inhibition can revert this phenomenon and stabilize the GJ-Cx43 junction increasing melanoma susceptibility to NK cell killing [189].

## Autophagy shapes NK and T cell trafficking in tumors

TME exerts a prominent role in tumor progression, tumor metastasis, and immunotherapy resistance. Different classes of cytokines (TNFs, IFNs, ILs, chemokines) are implicated in the regulation of cellular cross-talk and tissue homeostasis. Cytokines are soluble factors secreted in TME by different cell types (e.g., mesenchymal and epithelial cells) and contribute to cancer development based on their concentration into the tumor bed. Cancer cells are able to generate an immunosuppressive TME through cytokine secretion (e.g., TGFβ, IL-10) modulating immune cell activity and recruitment. Increasing the NK infiltration into TME represents the final point to render the current treatment for solid tumors more efficient. Autophagy manipulation is reported to be able to increase tumor-infiltrated NK cells. Autophagy inhibition, indeed, increases the sensitivity of solid tumors (e.g., melanoma and CRC) to ICIs by enhancing tumor-infiltrating tumor T cells (both CD8^+^ and CD4^+^) as well NK cells and other innate immune cells (e.g., DCs, M1 macrophages) in immunocompetent mice [166, 190]. Of interest, this does not happen in immunodeficient murine models, indicating the relevance of hot immune TME for the efficacy of autophagy blockade combined with ICIs in the treatment of solid tumors. Interestingly, in melanoma-bearing mice, after genetic inhibition of *Vps34*, it was possible to observe an increased tumor growth after NK cell depletion but not after CD8^+^ T cell depletion, suggesting the main role of NK cells in the anti-tumor response in the case of autophagy blockage [166]. Additionally, autophagy machinery has been also implicated in both chemokines and cytokines secretion [191] even if the exact mechanisms that govern this crosstalk have not been fully clarified. In the context of cancer, autophagy inhibition is reported to enhance immune cell tumor infiltration by increasing the secretion of pro-inflammatory IFNγ and C–C motif ligand (CCL)-5 (alias RANTES) and CXCL10 chemokine in melanoma, CRC and GBM murine models [166, 190, 192]. Silencing of CCL5 in *Beclin 1* KO melanoma cells reverts the ability of autophagy inhibition to regulate tumor regression and NK infiltration [190]. Moreover, *FIP200* inactivation in mammary tumor cells led to the increased CD8^+^IFN-γ^+^ and CD4^+^IFN-γ^+^ T cells by increasing CXCL9 and CXCL10 production [193]. Different reports tried to elucidate how autophagy blockage could induce chemokine secretion in cancer cells. *Beclin1* silencing in melanoma cells impairs the catalytic activity of Protein Phosphatase 2A (PP2A) on the JNK [190, 194, 195]. In this way, JNK triggers phosphorylation of the c-Jun transcription factor inducing *CCL5* mRNA expression [190]. Additionally, Vps34 targeting is associated with increased chemokine secretion due to STAT1/IFN-regulatory factor (IRF) 7 signaling pathway activation [166, 196].

## Potential clinical application for adoptive cell-therapy

From the observation that tumor cells are capable of modulating autophagy to promote their survival and resistance to current clinical treatments (radiotherapy and chemotherapy), studies about autophagy in cancer have expanded dramatically [197, 198]. On the basis of these studies, few novel autophagy inhibitors have been identified which have been then tested in human clinical trials. These preliminary results demonstrated that the efficacy of these drugs is still suboptimal and therefore new, more potent, and specific inhibitors should be explored [197].

One of the most important results obtained in the last two decades in the fight against cancer is represented by the development of immunotherapy approaches, strategies capable of redirecting cells of the immune system toward cancer cells. In particular, Chimeric Antigen Receptor (CAR) immunotherapies [199] have revolutionized biomedicine with new treatments for diseases for which there is currently no cure [200, 201]. However, tumor cells were demonstrated to be able to evade and develop resistances also in this scenario. Heng et al*.*, in fact, showed that in B-cell malignancies, upregulation of autophagy genes (*ATG3, BECLIN1, RB1CC1*) protected cancer cells from CD19 CAR T cell-mediated cytotoxicity. This occurs mainly through inhibition of the TNFα-induced apoptosis pathway and using autophinib, a Vsp34 inhibitor, significantly enhancing the killing effect of CAR T cells [202]. This is in line with a retrospective study investigating the tumor resistance mechanism after immunotherapy where the authors found that autophagy limits TNFα dependent activation of caspase 8 without modulating NF-κB pathway activity. However, the authors reported as well that this effect could be restored by the genetic inhibition of FIP200 [203]. Similar results were also reported by Shen et al*.* who performed a genetic screening on several cancer cell lines treated with Bispecific T-cell engagers and revealed that a change of autophagy-related genes is involved in their limited anti-tumor effect [204]. They and others also demonstrate how autophagy pathways could be shaped by tumor cells following therapies, for example, predisposing the generation of a cold tumor microenvironment [205–207].

Based on all these observations, it is now intuitive to wonder, what happens to immune cells used in these immunotherapeutic approaches? What does their autophagic flux look like? Would it be possible to modulate it like tumors do to improve their functionality and persistence? In this regard studies are still very limited and performed in the majority of the cases in murine models. However, based on these data, we can speculate that autophagy modulation may offer many benefits for patients treated with CAR T cells or other adoptive cell therapies. It has been reported that autophagy is present at a basal level in both circulating T and NK cells and upon activation autophagy is increased to support the new metabolic demands. However, low autophagy is observed in effector function T cells and it is associated with an increase in glycolysis, mitochondrial respiration inhibition, reduced ATP production, and decreased FAO levels. These phenomena are not always advantageous, especially from the perspective of long-term anti-tumor efficacy [208, 209]. In fact, autophagy inhibition may affect the number of apoptotic proteins and cell cycle inhibitors compromising survival and proliferation of adoptive cell products. Differently, autophagy induction could promote an increase of OXPHOS and FAO, which could help adoptive cellular products to differentiate toward a memory phenotype and increase their survival and proliferation by destroying pro-apoptotic proteins and cell cycle inhibitors. Then, it is now well known that the TME represents a physical and nutrient-deficient barrier for the adoptive cell infiltration and function. Therefore, a boosting of autophagy in these cells may improve their fitness and survival in the TME [210]. In order to identify more effective intervention strategies, we believe that we must not only focus on single approaches but also explore combined treatments in search of possible synergies. Given the role of autophagy inhibition in remodeling the TME and promoting immune cell chemokine secretion [193, 211], specific tumor autophagy inhibition may facilitate adoptive cellular product trafficking within the tumor masses and increase tumor-associated antigen expression.

Another example is reported in a study where the autophagy blockade in GBM murine models increased (CAR)-NK cell homing and cytotoxic activity [199]. In particular, autophagy inhibition via chloroquine treatment or Beclin1 silencing in patient-derived GBM cells was able to enhance NK cell tumor infiltration and tumor eradication in vivo. Additionally, autophagy inhibition enhances chemokine secretion (CCL5 and CXCL10) into the tumor bed. The authors demonstrated that targeting autophagy is sufficient to modulate NK cell-based immunological antitumor response in the GBM xenograft model. Moreover, the combined treatment of chloroquine and armored GD2.CAR-NK cells could counteract NK cell metabolic dysfunction caused by the TME [192].

## Conclusions

In conclusion, this review underlines the crucial role of autophagy in both T and NK cell development and function using both human and mouse models (Table 1).

**Table 1:** Summary of autophagy roles in both T and NK cells in human and other organisms

	**Evidence obtained from:**		
Topic	**Human cells**	**Murine cells**	**Other species**
Autophagy during T cell differentiation	[10]	[11–13, 15–17]	
Autophagy in regulatory T cells (Treg)	[20, 23, 24, 27, 28]	[18–22, 24, 28]	
Autophagy in CD4^+^ T helper cells	[32, 35]	[22, 29, 30, 32–34, 36, 37]	
Autophagy in CD4^+^ and CD8^+^ memory T cells formation	[41, 43]	[38–43]	[39]
Autophagy upon T cell activation	[41, 43, 47, 63, 64, 66, 67]	[21, 40–43, 48, 49, 51, 53–67]	
Autophagy and generation of energy upon T cell activation	[63, 69]	[34, 48, 63]	
Relationship between cytokines release and autophagy in T cells	[32, 63, 64, 72]	[21, 22, 32, 40, 42, 48, 49, 51, 55, 62–64, 73–75]	
Autophagy and T cell homeostasis		[53, 55, 57]	
Impact of mitophagy on T cell survival and homeostasis	[63, 69, 72, 79, 83, 85, 87, 89, 90]	[21, 30, 48, 53, 55, 56, 58, 63, 80, 84, 86–88, 90]	
Autophagy and ER in T cell survival and homeostasis	[66, 92, 99, 100, 103]	[40, 54, 66, 93, 94, 96–100]	
Impact of autophagy on T cell survival and cell death	[106]	[30, 49, 106]	
Autophagy in NK cell differentiation	[126, 128, 130]	[123–128, 130, 131, 133, 134]	[129]
Mitochondria-related processes in NK cells	[114, 137, 139, 140, 142–144, 146]	[124, 134, 138, 146]	
Autophagy in natural killer T cells (NKT)	[150–152]	[21, 36, 148–153]	
Autophagy at the interface between cancer cells and NK/T cell effector functions	[159, 160, 162, 167]	[154, 159–162, 166, 168]	
Hypoxia-induced autophagy in cytotoxic T and NK cell activity	[170, 181–185, 189]	[154, 174–176, 178, 182, 184, 187]	
Autophagy shapes NK and T cell trafficking in tumors	[190, 192, 195]	[166, 190]	
Potential clinical application	[192, 203, 204, 211]	[193, 203, 204, 211]	

This process is fine-regulated and dynamic since it is affected by various positive and negative feedbacks produced within the cells but also present in the microenvironment where these lymphocytes differentiate and work.

It is clear from the studies conducted so far that autophagy is a link between T and NK cell signaling and their metabolic processes in response to activation. As both T and NK cells are emerging as excellent candidates for adoptive immunotherapy approaches and the important results obtained with their redirection with CAR molecules [200, 212], the modulation of their autophagy machinery could represent a new strategy to overcome their actual limitations in implementing their persistence and functionality while reducing exhaustion [213–215]. However, many aspects of autophagy and autophagy manipulation in primary human T/NK cells are still unknown and require further investigation in view of possible clinical applications.

## Data Availability

Not applicable.

## Abbreviations

ADCC: Antibody-dependent cellular cytotoxicity
ADCD: Autophagy-dependent cell death
AIF: Allograft inflammatory factor
AMBRA1: Activating molecule in Beclin1-regulated autophagy
AMPK: AMP-activated protein kinase
AP-1: Activator protein 1
APC: Antigen-presenting cells
ATF6: Activating transcription factor 6
ATG: Autophagy related genes
ATP: Adenosine triphosphate
Bak: Bcl-2 homologous antagonist/killer
Bcl-2: B-cell lymphoma 2
Bcl-Xl: B-cell lymphoma-extra large
Bcl10: B-cell lymphoma/leukaemia 10
BIM: Bcl-2 Interacting Mediator of cell death
BM: Bone marrow
BNIP3: BCL2 interacting protein 3
BNIP3L: BCL2 interacting protein 3 like
CAR: Chimeric antigen receptor
CCl: C-C motif ligand
CCRC: Clear cancer cell carcinoma
CDKN1B: Cyclin-dependent kinase inhibitor 1B
CLPs: Common lymphoid progenitors
CMA: Chaperone-mediated autophagy
CO: Carbon monoxide
CRC: Colorectal cancer
cTECs: cortical thymic epithelial cells
Cx43: Connexin 43
CXCL8: C-X-C motif chemokine ligand 8
DCs: Dendritic cells
DN: Double negative
DNAM-1: DNAX accessory molecule 1
DP: Double positive
DRP1: Dynamin-Related Protein 1
ECAR: Extracellular acidification rate
FR: Endoplasmic reticulum
ER-phagy: Endoplasmic reticulum autophagy
ERQC: Endoplasmic reticulum quality control
FAO: Fatty Acid Oxidation
FasL: Fas Ligand
FIP200: Focal adhesion kinase interacting protein 200 kDa
Fox: Forkhead Box
GBM: Glioblastoma
GJ-Cx43: Gap-junctional connexin 43
GLUT1: Glucose transporter 1
HIF-1α: Hypoxia-inducible factor 1α
HMGB1: High mobility group box 1
HSCs: Hematopoietic stem cells
ICIs: Immune checkpoint inhibitors
IFNγ: Interferon γ
IL: Interleukin
IL-7Rα: Interleukin-7 receptor subunit alpha
ILCs: Innate lymphoid cells
iNKs: Immature NKs
iNKT: invariant NKT
IRE1α: Inositol-requiring enzyme 1α
IRF: IFN-regulatory factor
Itch: Itchy E3 ubiquitin protein ligase
JNK: Jun kinase
L-Arg: L-arginine
LAMP-2A: Lysosome-associated membrane protein 2 isoform A
LC3I: Lipidated microtubule-associated protein 1A/1B light chain 3
LC3II: LC3I+PE
LKB1: Liver kinase B1
miRNA: Micro ribonucleic acid
mNK: Mature NK cells
mRNA: Messenger ribonucleic acid
mTECs: medullary TECs
mTOR: Mammalian target of rapamycin
mTORC: Mammalian target of rapamycin complex
NAD: Nicotinamide adenine dinucleotide
NF-κB: Nuclear factor-κB
NFAT: Nuclear factor of activated T cells
NK: Natural killer
NKPs: NK cell Precursors
NKR: NK-activating receptors
NKR2: NK-activating Receptors
NKT: Natural killer T cells
NLRP3: NLR family pyrin domain containing 3
NR: Nicotinamide riboside
NSCLC: Non-small cell lung cancer
OCR: Oxygen consumption rate
OPA1: Dynamin-like 120 kDa protein, mitochondrial
OXPHOS: Oxidative phosphorylation
OXPHOS: Oxidative phosphorylation
PARKIN: Parkin RBR E3 ubiquitin-protein ligase
PCD: Programmed cell death
PE: Phosphatidylethanolamine
PERK: Protein kinase R-like endoplasmic reticulum kinase
PERK: Protein kinase RNA-like ER kinase
PI3K: Phosphoinositide 3-kinase
PIK3C3: Phosphatidylinositol 3-kinase catalytic subunit type 3
PINK1: PTEN-induced kinase 1
pMHC: Peptide-loaded major histocompatibility complexes
PP2A: Protein Phosphatase 2A
PTPN1: Protein-tyrosine phosphatase 1B
PTPN1: Protein Tyrosine Phosphatase Non-Receptor Type 1
Ras7: Ras-related protein
RCAN1: Regulator Of Calcineurin 1
ROS: Reactive Oxygen Species
RPS6KB: Ribosomal protein S6 kinase beta
RPS6KB (p70): Ribosomal protein S6 kinase B 70 kDa
SP: Single positive
STAT3: Signal Transducer and Activator of Transcription 3
STING: Stimulator of interferon genes
TAX1BP1: Tax1-binding protein 1
TCR: T cell receptor
TFEB: Transcription factor EB
TGFβ1: Tumor Growth Factor β1
TILs: Tumor-infiltrating T lymphocytes
TME: Tumor microenvironment
TNFα: Tumor Necrosis Factor α
TRAIL: Tumor Necrosis Factor-Related Apoptosis-Inducing Ligand
Treg: Regulatory T cells
ULK: Unc-51-like kinase
UPR: Unfolded Protein Response
XBP1: X-box binding protein 1
XIAP: X-linked inhibitor of apoptosis protein
